# Driving for Success in Family Reunification—Professionals’ Views on Intervention

**DOI:** 10.3390/ijerph192416594

**Published:** 2022-12-10

**Authors:** Diana N. Teixeira, Isabel Narciso, Margarida R. Henriques

**Affiliations:** 1 Center for Psychology at University of Porto (CPUP), Faculty of Psychology and Education Sciences, University of Porto, 4200-135 Porto, Portugal; 2 Research Center for Psychological Science (CICPsi), Faculty of Psychology, University of Lisbon, Alameda da Universidade, 1649-013 Lisboa, Portugal

**Keywords:** family reunification, professionals’ views, intervention influential factors, (un)successful trajectories, qualitative approach

## Abstract

Family reunification is a complex process and is consensually considered the best solution for children in care, as soon as the family has changed the dysfunctional patterns that prevent child safety and well-being. Intervention throughout the entire process is crucial to the success of family reunification. This study aimed to explore and understand child protection professionals’ views on factors influencing (un)successful family reunification trajectories. Using a qualitative design, 33 Portuguese child protection professionals participated in five focus groups. The thematic analysis revealed a set of influential factors within three different systemic levels: child, family, and child welfare system. The latter level was clearly predominant, pointing to the powerful role of the intervention as a vehicle for successful family reunification. The results showed the relevance attributed by the professionals to some main intervention guidelines, children–professionals’ relationships, multisystemic assessment and intervention, coordinated work of intervention teams, and sufficient time between the court decision and the child’s re-entry into the family home. The need for early intervention and its continuity after the child’s reintegration into the home also emerged as relevant factors. This study provides in-depth knowledge of professionals’ views on the intervention process, thus contributing to a comprehensive understanding of (un)successful family reunification trajectories.

## 1. Introduction

Family reunification occurs when children return to their biological family following a period of removal from their families to foster care [1]. According to the Children’s Bureau [2], the decisions to remove children from their families and place them into the foster care system are commonly taken when their safety cannot be assured, which is usually associated with physical abuse, sexual abuse, and negligence.

Although children’s removal from their families is justified by the urgency of their protection and security, this event and their entry into institutional care have a detrimental impact on their well-being and psychological adjustment. Following their removal, most children display externalization and internalization behaviors [3,4] and insecurity and greater disorganization in their attachment relations [5,6,7], and this has a highly negative impact on their life [8]. Following removal from the family and during their placement in foster care, children have to cope with the emotional impact triggered by multiple losses as well as with the need to adapt to new contexts [9]. Additionally, they have to deal with the challenge of choosing to be loyal to their biological family or foster family [10]. Similarly, the family has to cope with their own emotions triggered by the child’s removal and face the negative social perception of the event and the social judgment of being unqualified families for parental performance [11]; moreover, they have to deal with all the challenges related to the child’s placement, which results in a significant increase in stress [12,13].

Family reunification as a Life Project is consensually considered the best solution for children in care, as soon as the family dysfunctional patterns that hindered the child’s safety and well-being are changed to balanced patterns [14,15]. Previous research has shown that long periods in social care are associated with loss of family connections and identity and with difficulties in re-entry into the family [14]. Balsells et al. [16] report that children experience mixed feelings related not only to their sense of belonging to the family, but also to rejection, abuse, and abandonment, often resulting in a complex reunification process experienced with considerable ambivalence. Furthermore, following their reintegration into the family, children and their families face many challenges and demands in the process of renewing family bonds, identity, and functioning that may compromise their mutual re-adaptation [17]. In this regard, several authors have highlighted the pressing need for intervention with both the family and children, not only during the foster care period, but also in the transitions from home foster placement and from foster placement to home, as well as in the post-reunification phase [17,18,19,20,21,22,23].

The knowledge regarding the family reunification process is clearly insufficient, particularly in Portugal. An in-depth understanding of this process could increase the success of reunification and contribute to the well-being and protection of children; moreover, research on the family reunification process is often based on information collected from databases or children’s records [19,24,25,26,27,28]. However, to further enrich professional practices, a comprehensive understanding of the reunification process is necessary, which also requires direct access to the perspectives of the professionals who work with children and their families within the foster care system [22]. Thus, the present study seeks to explore and understand child protection professionals’ views on the factors influencing (un)successful family reunification trajectories.

### 1.1. The Multiplicity of Risk Factors in the Family Reunification Process

The child’s re-entry into foster care due to the recurrence of danger (e.g., child abuse, severe neglect) may be viewed as an unsuccessful family reunification process [29,30,31,32,33]. Re-entry into foster care has a lasting negative impact on the child [34], particularly regarding attachment and emotional and behavioural stability [18].

The scientific literature has already identified several factors with a negative impact on the success of family reunification, which are frequently classified into three groups: child characteristics, family characteristics, and child welfare service characteristics. With regards to the child-related characteristics that have a negative impact on the success of reunification, the scientific literature highlights children’s emotional problems, externalizing behaviours, and mental health problems [21,29,30,35,36,37]; attachment issues [38]; special needs due to physical problems [29,35]; cognitive difficulties [21]; children’s exposure to illicit substances in the prenatal period [29]; and the age of the child, particularly infants, pre-teens, and adolescents [24,28,35,37,39].

As far as family-related factors are concerned, prior empirical research points to neglect as grounds for removal [24,29,36,40]; parents’ lack of motivation for family reunification [29,40]; unpredictable changes in the family composition or specific life circumstances [18,29,31,36]; parents’ history of substance abuse [28,29,35]; domestic violence and parents’ history of traumatic events [35,38]; low socioeconomic status [29,35]; lack of social support [29]; and the high number of early identified family risk factors [24,35]. Some authors have also underlined the existence of numerous children [29,35] and other siblings in foster care [29,30].

Concerning child-welfare-system-related factors, the empirical literature emphasizes the length of time in care [21,24,29,30,40]. Some empirical studies have revealed that overly short periods in foster care—less than six months—do not provide sufficient time for effective intervention [24,29,30]; furthermore, longer periods—over 12 months—present a risk factor for reunification, which increases the longer the child is in care [21]. The literature also points to several other factors deemed detrimental to the success of the reunification process: recurrence in foster care [27,30,37,41]; inconsistencies in assessment and intervention practices [18]; inadequate preparation of children and parents for reunification [18,19,28,33]; poor communication between professionals and family, which discourages parental involvement [18,42]; a large number of foster placements [29]; lack of the development and implementation of predictive risk assessments for re-entry [21]; intervention restricted by tight deadlines in order to accelerate the reunification process [21,41]; returning to the family without sufficient professional support [18,31,35]; inadequate practices after reunification [18,26] and no contact between the professionals and family after reunification [30,43]; failures regarding decision-making [18]; and court decisions to reunify against the opinion of the professionals [18,30]. Some authors have also emphasized the family’s need for support from the protection system prior to the child’s removal [24,29] and the child’s placement in residential care compared to family foster care, which can have a negative impact on successful reunification [27,29].

Such a multiplicity of risk factors, as well as the unpredictability of the positive changes being maintained in the family system, do not provide a precise indication of whether reunification will be successful; however, family preparation [18,19,44] and child preparation [20] for family reunification are regarded as essential for successful family reunification.

### 1.2. Intervention in the Reunification Process

Intervention is crucial to change dysfunctional patterns and to prepare children and families to constructively cope with the multiple challenges during this process, which is also characterized by uncertainty [20]. According to the Child Welfare Information Gateway [45], the reunification intervention should encompass three stages: preparing the family (including children) for reunification, providing intensive professional support after the child’s return to the family home, and providing follow-up.

Given the toxic impact of multiple highly adverse factors (e.g., previous experience of maltreatment, the violent moment of removal, the temporary loss of the family and their daily context) on children, the preparation for return to the family is imbued with considerable complexity [12]. Several authors argue that preparation for reunification should begin early, soon after the child has been removed from the family [20,23,29,30,31,43] and prolonged after returning to the biological family [18,20,39].

The process of preparing for family reunification includes an initial comprehensive assessment phase [14,20,40], which is essential to identify risk factors—and particularly child and family weaknesses and strengths—in order to delineate individualized family intervention [46]. According to the *Framework for the Assessment of Children in Need and Their Families* [47], based on Bronfenbrenner’s [48] bioecological model, assessment and intervention should consider the following as central axes: (1) the child’s developmental needs; (2) parenting skills to respond to the child’s needs; and (3) family and contextual factors.

The family’s preparation for reunification before the child returns home is essential for modifying dysfunctional family patterns and for the consolidation of appropriate parenting, as it provides a systematic interventive focus on specific child and family needs (e.g., parental role definition, parenting skills, attachment patterns, realistic expectations, reinforcement of family participation in the child’s life, social support) as well as a focus on parental self-efficacy and self-confidence and family identity [49,50].

Following reunification, support for the children and their families should be ensured for the initial months, at least through a post-reunification service [18,20,21,26,29,43,45], as this is the period in which relationships and bonds are re-established [30]. Font et al. [21] add that commitment to continued support after reunification also offers greater security to the reunification decision. Although post-reunification support entails increased financial costs at an initial stage, when used consistently it brings long-term economic gains due to the positive effect on the reunification’s success [18,38]. Despite this evidence on the importance of post-reunification intervention, in Portugal there is neither a clear and formal indication for the provision of post-reunification support for all families, nor is this legally established in the law.

The support provided after reunification should be gradually reduced to become a follow-up support in accordance with the family’s needs [18]. These follow-up periods can contribute to the success of family reunification [41,43] and afford a smooth transition as well as stable reintegration [51].

During the intervention in the reunification process before the child returns home, professionals are confronted with several challenges due to the complexity of this process [12]. Failures in decision-making can contribute to unsuccessful family reunification, which leads to insecurities that are intensified by the lack of working guidelines [18]. These insecurities, the tight deadlines, and, in some cases, having to work against professionals’ own opinions due to court orders, contribute to an environment of pressure at work [18,30,52]. Interprofessional communication and collaborative relations can contribute to mitigating these pressures, allowing for the creation of feasible intervention plans, thereby making it possible to identify and address the barriers to reunification and to define each professional role [25,30,53,54]. Arbeiter and Toros [55] argue that training and experience in reunification promotes greater knowledge and the consequent effectiveness and confidence of professionals in their work. Finally, the use of professional supervision and reflection on practices with other professionals support decision-making during family reunification processes and contribute to the quality of work [18,20].

### 1.3. The Present Study

The present study aims to explore and understand the perspectives of Portuguese child protection professionals regarding the factors that influence the process of family reunification. Recent Portuguese data on foster care [56] show that about 6706 children have left the care of their parents or guardians and have been placed under the custody of Child Protective Services. Most of them were placed in residential foster care (86%), while a minority (3%) were placed in family foster care. The decision to remove children from their families and place them into the foster care system is commonly taken when their safety cannot be assured. The most common reasons for foster care placement are negligence (71%), as well as other situations, such as temporary lack of family support, abandonment or deviant behaviour (13%), psychological maltreatment (10%), physical maltreatment (4%), and sexual abuse (2%). In Portugal, family preservation is the core goal of the child welfare system, which means that family reunification is considered the best solution once the family has changed the dysfunctional patterns that hindered the child’s safety and well-being. As soon as the child is placed under the custody of Child Protective Services, there is an initial assessment period that aims to design their Life Project (e.g., family reunification, adoption, autonomy process) that is legally defined by the court. Once the family reunification Life Project is defined, social care professionals begin the process of intervention with the family and the child to carry out a successful homecoming. Notwithstanding the excessively long duration of foster care (on average more than 3 years) and the high number of unsuccessful family reunifications with re-entry into foster care [56], the scarcity of empirical research to examine the strengths and handicaps of the intervention process as well as its impact on (un)successful family reunification should be noted. As such, the present study aims to analyze the experiences and perspectives of Portuguese child protection professionals (psychologists, social workers, and educators) regarding the reunification process, as their voices are less commonly considered for understanding this phenomenon. Thus, using a thematic analysis methodology, this study aims to respond to the following research question: Considering the practice of child protection professionals, which main factors contribute to (un)successful family reunification trajectories?

## 2. Materials and Methods

### 2.1. Participants

The study consisted of 33 participants distributed across 5 focus groups: 3 groups with technical workers, namely professionals with case manager duties in residential care and professionals in specialized teams who focus on preservation or reunification work with the family (G1: *n* = 10; G2: *n* = 3; G3: *n* = 9); and 2 focus groups with educational workers, namely professionals with daily childcare duties on residential care, such as waking up the children, giving them a shower, or helping them with schoolwork (G4: *n* = 7; G5: *n* = 4).

The participants were selected from teams working in residential care, which is the main response for children removed from their families in Portugal, or directly with families whose children were in residential care. In our country, it is also customary for residential care to include two teams of professionals: technical workers (with case management responsibilities) and educational workers (with daily routine support duties). These professionals, sometimes working in situations of family reunification, are supported by other teams that specialize in working with families.

The set of participants included 14 professionals with technical duties in residential care, 8 professionals with technical and specialized duties in working with families, and 11 professionals with educational duties in children’s daily lives in residential care. All the professionals—13 psychologists; 8 social workers; 4 social educators; 2 early childhood educators; 1 sociocultural animator; 1 humanitarian educational worker; 1 sociologist; and 3 professionals without higher education—were working in the Portuguese system for the promotion and protection of at-risk children and young people. They were predominantly female (*n* = 30; 90.1%), aged between 26 and 56 years old (*M* = 38; *DP* = 6.5), and had from 1 to 32 years of work experience in this field (*M* = 11.5; *DP* = 6.14). They were from two main urban regions of Portugal; namely, 16 professionals were from Porto in the North Region and 17 were from Lisbon in the Central Region of the country. It should be noted that these regions are core centers of the Portuguese system for the promotion and protection of at-risk children and young people.

### 2.2. Data Collection

The data were collected through five focus groups, as this is an effective method of qualitative data collection for exploring subjective experiences, meanings, and processes [57]. This enabled access to the participants’ perspectives and beliefs, as well as use of the interaction between them to encourage sharing. The focus groups lasted an average of 102 min, ranging from 65 to 135 min, and all of them were conducted by the same researcher, the first author of this paper.

Aiming to sample ideas rather than people to generalize the results, as is recommended within qualitative approaches [58,59,60], a convenience sample was used, which was guided by the objectives and namely comprised professionals from teams with over six months of professional experience. In line with qualitative approaches, the number of focus groups was defined as a function of the approximation to the theoretical saturation point, meaning that the inclusion of new data did not add relevant information [59].

Initial contact was made with the directors of several institutions to present the objectives of the study, as well as the conditions and procedures of the interviews, namely the need for video recording, the voluntary participation of professionals, and the guarantee of confidentiality. The directors shared this information with their staff in order to make an informed decision regarding their voluntary and free participation.

The focus groups were held in the most convenient locations for the participants. Three were held at the institutions’ head office and two at the university where the researcher works. Before beginning the interview within the focus group, the researcher provided detailed information on the objectives of the study, the procedures during the interview, the need to video record for an in-depth analysis, and also ensured confidentiality of the data, which was followed by completion of the informed consent and the form containing the sociodemographic data.

The study protocol was approved by the Ethics Committee of the Faculty of Psychology and Educational Sciences of the University of Porto.

### 2.3. Instruments

Two interview scripts were created, one for the focus groups with professionals from technical teams, and another for the focus groups with professionals from educational teams. The script for the focus groups with technical teams included four central themes: intervention practices with children and family; needs and difficulties of their work; perspectives on factors that may influence the success or failure of family reunification; and views on the importance of preparing the child—e.g., “We would like to start by getting to know your practices. What do you do with the child and/or family in a situation where family reunification is expected?” and “In the process of family reunification, what needs and difficulties have you identified while performing your work?”.

The script for the focus groups with educational teams included six central themes: their involvement in intervention practices on preparing for reunification with children and parents; perspectives on how children regard the family reunification; well-being indicators of the relationship between children and parents, observed through family meetings; impact of a child leaving residential care for family reunification on the foster home; existence of a relationship between care professionals and children/parents following family reunification; and perspectives on the importance of preparing the child for reunification—e.g., “On the basis of your experiences, when are children prepared for family reunification?” and “What indicators allow you to ascertain whether the children’s well-being is assured before and after family visits?”.

A questionnaire was also prepared to collect participants’ sociodemographic data, such as age, gender, and information regarding their professional trajectory—such as training and length of professional experience in the area.

### 2.4. Data Analysis

The qualitative analysis was accomplished through an inductive process focused on the personal and subjective perspectives of the participants, although previous theoretical frameworks were also taken into consideration, thereby allowing for a systematized and in-depth understanding of the phenomenon under study [61]. A thematic analysis was conducted to identify patterns and recurring themes from the perspectives of the participants [62,63]. To this end, all the interviews were audio-recorded and transcribed verbatim by three members of the research team. The transcripts were reviewed by the principal investigator in order to identify the transcribers’ potential misunderstandings before initiating the data analysis.

QRS Nvivo 12 software was used to support the qualitative data analysis, which was conducted according to the procedures recommended by Braun and Clarke [62]. Therefore, the main researcher first invested in becoming acquainted with the data by reading all the transcripts and making notes of the initial ideas. He then coded the relevant contents of the data, relating them to categories created at the same time. These categories were grouped into meaningful themes that were later revised to ensure they were in line with the coded extracts. Finally, the main researcher reviewed and clarified the name of each theme, aiming to enhance coherence.

It should be noted that two senior researchers with specialized knowledge in qualitative analysis continuously supervised and discussed the entire research process, contributing to increased reflectivity and reducing the influence of subjectivity.

## 3. Results

The qualitative analysis revealed several influential factors on the family reunification trajectory, which were organized into three different systemic levels according to the classification commonly used in empirical studies in this field: child, family, and child welfare system [33].

Considering the three systemic levels, nine main themes were defined (Table 1) that were related to key issues that foster the success of family reunification: *Child’s Autonomy*; *Disclosure of Expectations*; *Family Stability*; *Parent–Child Affective Bonds*; *Guideline Hotspots for Intervention*; *Children–Professionals Relationship Quality*; *Multisystemic Assessment and Intervention*; *Functionality and Coordination of Intervention Teams*; and *Sufficient Time between the Reunification Decision and the Child’s Re-Entry into the Family Home*.

It should be noted that the participants’ quotes were identified by a letter representing the participants’ professional duties (ED—Educational Duties; TD—Technical Duties, i.e., Psychological or Social Work), followed by letters identifying the locus of intervention (IF—intervention with the family; IRC—intervention in residential care), the participant’s age (e.g., 40), and, finally, identification of the focus group (G1, G2, G3, G4, G5).

### 3.1. Child-Related Factors

The child-related factors that emerged during the discourse of the participants—*Child’s Autonomy* and *Disclosure of Expectations*—were mentioned less than family-related and child-welfare-system-related factors.

#### 3.1.1. Child’s Autonomy

According to the professionals, primarily those with educational duties, the reunification process benefits when children are more autonomous, which was a trend that emerged when associated with more maturity—“the success of family reunifications depends a lot on their [children] characteristics, for example, with a very needy child it is more difficult than with a more mature child who is able to be alone” (ED; IRC; 36; G4).

#### 3.1.2. Disclosure of Expectations

The child’s disclosure of expectations regarding the future with their families emerged as a relevant factor for reunification success. The narrative of some participants showed that talking about these expectations with the child made it possible to regulate misadjusted expectations, which could unbalance the success of the child’s return to the family—“it is better that they [children] express expectations for the future so that we can work with them” (TD; IRC; 40; G1).

### 3.2. Family-Related Factors

The qualitative analysis of the professionals’ discourse revealed two main themes—*Family Stability* and *Parent–Child Affective Bonds*. Almost all the participants clearly evidenced a negative family view, pointing to their weaknesses—“I think these families have always had many weaknesses and continue to have them even with the support we provide” (TD; IRC; 40; G1).

#### 3.2.1. Family Stability

The *continuity in the family structure*, the *maintenance of the family as a priority*, and the *stability of the family social network* emerged as relevant factors that influence the success of family reunification.

According to the experience of most professionals, the success of family reunification is jeopardized by the frequent departures or entries of nuclear family members:

“I think that the separation from her partner [another relationship after separation from the child’s father] was a turning point in the whole process: the mother began to have a negative relationship with her partner and decided to break up to start a new relationship... the children were still at home [with their mother], but throughout this process of people coming in and out, they were taken from her again.” (TD; IRC; 39; G2).

Most of the participants referred to the parents’ ability to prioritize family needs, goals, and projects as being highly relevant for successful family reunification—“She [the mother] redefined her priorities and started to invest more in her dream job, and the children were no longer a priority” (TD; IF; 37; G2).

The family difficulty of maintaining social relationships over time was also highlighted by the professionals in the technical teams. In their view, the strategic work developed with the families to reinforce their social support is often impaired by the relational instability within social networks—“A neighbour [of the family] used to turn up during the visits and we welcomed her. We invested in this, but in the meantime the family fell out with the neighbour and this work was lost” (TD; IRC; 43; G3).

#### 3.2.2. Parent–Child Affective Bonds

According to all the participants, the emotional bonds between the parents and the child are crucial to the success of the reunification process, even if a close relationship characterized by fond love is not synonymous with a caring and supportive relationship—“when there is an emotional bond between the parents and children, we can work on the functional level, but when there isn’t, the process is far more difficult” (TD; IRC; 36; G1); “There has to be a connection foundation when everything else around the child is falling to pieces” (TD, IRC; 47; G1). In the same vein, almost all the professionals mentioned the importance of the parents’ ability to establish a strong affective bond with their children.

The negative impact on the quality of the relationship between parents and children due to their placement in foster care and, consequently, to less proximity, emerged as a detrimental factor to the success of family reunification

“I think it’s important that the family and child are not cut off from each other in the case of foster care placement. This would frequently happen, and the families would become detached from their children (…) A huge [temporal] gap between foster care and family reunification was also frequent. When there is a gap like that, if there isn’t a very close relationship between those responsible for the child [in residential care] and the family, they effectively become cut off from each other and the parents become detached from their children, and the children also become detached from their parents” (TD; IF; 37; G1).

Additionally, several professionals with technical duties highlighted that intervention should promote, reinforce, and protect the development of intrafamilial affective ties—“they [the families] must experiment, see each other, feel, be together again.” (TD; IRC; 43; G3); “We can only move towards successful family reunification if we indeed open the door to the experiences between parents and children.” (TD; IRC; 41; G1). It was also emphasized that increased parent–child proximity (e.g., visits, weekends at home, holiday periods at home) during the final phase of the reunification process enhances the development of affectivity between parents and children and, simultaneously, enables its assessment which, in turn, informs the intervention process—“Sometimes when they start having weekends [at home] and things don’t go well, there has to be an interruption. But if they didn’t go, we wouldn’t know whether it would work out or not. Obviously, an investment to repair this has to be made afterwards with the child, and this must happen” (TD; IRC; 39; G3).

### 3.3. Child-Welfare-System-Related Factors

**Figure 1:**
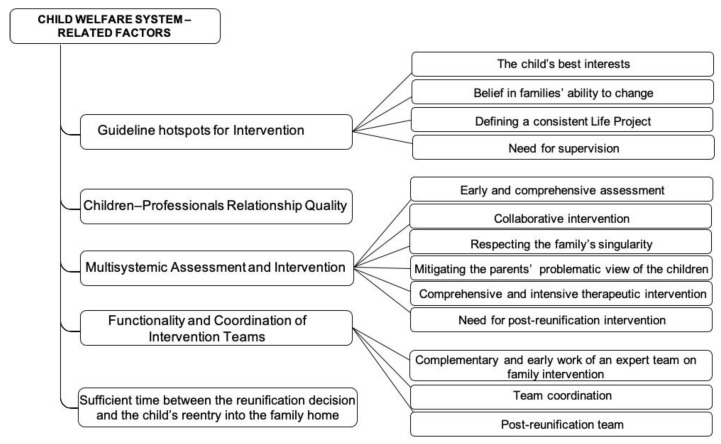
Child-Welfare-System-Related Factors—Thematic categories and respective subcategories with an influence on the family reunification process.

The qualitative analysis revealed five themes corresponding to multiple factors related to the intervention system with an impact on the success of reunification: *Guideline Hotspots for Intervention*; *Children–Professionals Relationship Quality*; *Multisystemic Assessment and Intervention*; *Functionality and Coordination of Intervention Teams*; and *Sufficient Time between the Reunification Decision and the Child’s Re-Entry into the Family Home*. These thematic categories include diverse subcategories, as shown in Figure 1.

#### 3.3.1. Guideline Hotspots for Intervention

This thematic category includes four subcategories: the child’s best interests, belief in families’ ability to change, consistent definition of the child’s Life Project, and need for supervision.

***The child’s best interests.*** The focus on the child as a priority emerged as a main principle to guide the entire intervention and decision process—“(…) it is essential to think about whether we’re talking about the family’s right to their child or the child’s right to a family. The child’s best interest must guide our work” (TD; IRC; 43; G3); “the principle is always: what the best for the child is. We have to guide ourselves on that to be successful” (TD; IRC; 47; G1).

***Belief in families’ ability to change.*** This subcategory was mentioned by some of the professionals in all the focus groups where the participants had technical duties. They highlighted that believing in families’ ability to change is crucial to successful reunification—“Someone who works with families has to believe in the intervention with families in order to make it work” (TD; IRC; 39; G3). Notwithstanding the intricate multiplicity of problems, the family system can evolve through intervention:

“We’re talking about families, therefore it’s not something static, it’s dynamic. It’s been six months, a year or more [referring to past events] and already some things are different. This perspective helps with the work with the family now and will bring them gains in the future” (TD; IRC; 38; G3).

***Defining a consistentLife Project.*** All the participants stressed the importance of designing a consistent Life Project for each child (i.e., a child life plan for the future that considers family reunification, adoption, or autonomy) that is designed and suggested by the care system technicians and legally defined by the court, and ideally also considers the interests of the child. According to the professionals, each Life Project should be coherently grounded on a solid and comprehensive evaluation of the child as this guides the specific nature of the intervention during the child’s period in the social care system—“It’s up to us to support a Life Project. (…) We have to find strategies to present the substantiated facts and so that the best Life Project for that child can be defined.” (TD; IRC; 47; G1).

***Need for supervision.*** The supervision of professionals emerged as a particularly relevant factor to increase the objectivity of the evaluation—“separating the rational from the emotional is sometimes complicated” (TD; IRC; 39; G3). According to the professionals of one focus group where the participants held technical duties, supervision also contributes to the intervention’s efficiency—“(…) supervision is essential for us to have an outside perspective (…) our supervisors help us reflect and this leads to more effective intervention on our part” (TD; IRC; 35; G3).

#### 3.3.2. Children–Professionals Relationship Quality

Most of the participants highlighted the importance of the quality of the relationship established between the professionals and the child during the period of foster care:

“These children already have an idea of what it’s like to be at home [referring to the negativity of the family relational environment], but they really want to return. In any case, they have to at least be able to trust someone for once” (TD; IRC; 56; G1). It was also stressed that the children should be acquainted with the professionals in the court support teams as they are the ones who will supervise the family after reunification to assure its success—“It’s important that the professionals of these teams are able to go to the homes and meet the children for the process to go well… to talk to them, spend time with them, because this is what will happen later and it makes things easier” (TD; IRC: 39; G3).

#### 3.3.3. Multisystemic Assessment and Intervention

Within this thematic category, the qualitative analysis revealed six different topics—*early and comprehensive assessment*, *collaborative intervention*, *respecting the family’s singularity*, *mitigating the parents’ problematic views of the children*, *comprehensive and intensive therapeutic intervention*, *and need for post-reunification intervention*.

***Early and comprehensive assessment.*** The analysis of the data highlighted the urgent need for a comprehensive assessment—“(…) it should start at the moment the child enters the foster home or prior to removal, when the child has been signposted” (TD; IF; 42; G2)-, thus allowing for complete and rich information regarding the family functioning, and the identification of key intervention areas—“for instance, parental capacity, the characteristics of the child in different contexts” (TD; IF; 39; G2); “an in-depth assessment is important to start [the intervention] and for what may come next (...) minimizes the risks” (TD; IF; 39; G2).

Many professionals also emphasized the idea that the comprehensive assessment process should never be limited to observation of family visits to the child when in foster care, as this will provide limited information that is by no means indicative of the family’s relational reality—“these [family visits] are restrictive, and then it’s difficult to generalize what happens in terms of these families’ actual ability to care for their children, even if it’s just at a functional level” (ED; IRC; 36; G4). They also stressed that alternative means should be found to gain more in-depth knowledge of the families, such as inviting and encouraging the families to participate in their children’s lives in the foster home.

***Collaborative intervention.*** Collaborative intervention with the family emerged as a highly relevant factor:

“There can never be intervention where imposition is involved (…) we must think together and share suggestions, because if we have an attitude involving imposition, we’re not going to have positive outcomes in this task… experience has taught us that. We don’t stop being technicians (…) but sometime, we have to be a bit on the same level (…) try to create a closer bond” (TD; IRC; 39; G3).

Likewise, the quality of the relationship between the family and the professionals emerged as a highly relevant factor for the development of a collaborative context and successful reunification—“I think it’s a protective factor when we manage to create a good relationship with the parents”; “the relationship established with the family is an indicator of success” (TD; IRC; 41; G1); “I think that from the moment we create a good relationship, a trusting relationship, everything else flows. It’s the basis” (TD; IF; 37; G2); “I think that in terms of collaboration what works really well is to make them see that this is an opportunity, we’re going to prove to the courts, to the services or to whoever may be that we’re going to have the required conditions” (TD; IF; 39; G2).

According to the experience of almost all the participants, the relationship alliance and the collaborative work between the technician and the family foster greater involvement and reliable bonds, which are resources for the family—even in the future, after the family has already been reunified—“Even when they [the children] are already integrated [within the family], the parents know that if they need help, they can call” (TD; IRC; 41; G1).

***Respecting the family’s singularity.*** Several participants revealed a critical position towards the performance of professionals in general and underlined the importance of respecting the family’s values—“(…) significant family. They often get here having been completely scorned and discredited because they weren’t understood” (TD; IRC; 43; G3);

“There’s a lot of prejudice on the part of professionals [towards the families]. We have our own values, and they often don’t correspond to those of the families. Therefore, we make a direct comparison and think that something is not right but if we consider their values, it is. We have to shift from that and respect them” (TD; IF; 37; G2).

By reinforcing the idea of acknowledging singularity, the need for personalized intervention with the family was stated, thereby considering the specific characteristics of the child in order to equip the families to respond to the needs of their child—“It’s necessary to provide these parents with strategies for the real child we have, to foresee what might happen, so that the family is equipped with strategies to be able to deal with the child. That is what’s essential for success” (TD; IRC; 36; G1).

***Mitigating the parents’ problematic views of the children.*** The discourse of some of the professionals reveals the importance of helping the family to perceive the child as a symptom of something that may be wrong in the family system, instead of regarding them as the problem, which may negatively influence the family’s relationships after reunification—“otherwise when the child returns, in their view [the family’s], the problem returns” (TD; IRC; 43; G3).

***Comprehensive and intensive therapeutic intervention.*** The analysis revealed a critical position of almost all the professionals, mainly with technical duties, towards the quality of their intervention, thereby highlighting the need for more extensive and intensive interventions—and ones that are not only geared towards responding to basic care issues and instrumental needs. The difficulties in improving the quality of the intervention were attributed not only to the technicians, but also to the families—“(…) perhaps there is not always an intensive strategic approach, with therapeutic purposes. Sometimes, it is simply monitoring the family for instrumental aspects” (TD; IF; 42; G2); “Although the professionals have more in-depth work (with the families) in mind (…) it’s hard to intervene in these dynamics [communication, relationship] (…) they only focus on goals such as having a bedroom, a job…” (TD; IF; 42; G2).

***Need for post-reunification intervention.*** The need to continue intervention with the family after reunification was underlined by all the participants—“we feel that, if there were regular psychological counselling [after family reunification], in some families things could work out differently” (TD; IRC; 39; G3). The families’ access to such intervention also emerged as a concern—“For that follow-up to occur, the families need the financial conditions and many [families] don’t have enough money to ensure that kind of treatment” (TD; IRC; 39; G3).

#### 3.3.4. Functionality and Coordination of Intervention Teams

The narratives of the participants revealed the following main topics: *complementary and early work of an expert team on family intervention*, *team coordination*, and *post-reunification team*.

***Complementary and early work of an expert team on family intervention.*** The support of a specialized team that works with families to complementarily intervene with the foster home team prior to reunification, during the foster care period, was considered beneficial by all the professionals:

“the results were more effective when [the intervention team specialized in the family] starts intervening with the family earlier, before the child is home (…) since there are situations (...) where there are still risks that weren’t identified and worked on” (TD; IF; 39; G2).

***Team coordination.*** All the professionals underlined the importance of cooperation between the foster home team and the intervention team with the family, particularly in the transition periods—“more cooperation is important in reunification processes. In a timely manner, at a given stage of the process, cooperating with the team that will take over after the transition” (ED; IRC; 36; G4), which facilitates the exchange of relevant information, the family’s acceptance of the new team, and, consequently, the development of a cooperative context—“It’s important the children don’t feel an abrupt cut, that they feel there’s a member or someone representing the team doing the monitoring, because they’ll struggle. That’s a fact!” (ED; IRC; 29; G4).

Most of the professionals also referred to the importance of a clear definition of the team’s and professionals’ roles—“when things are clear, the roles of each are defined, things go well” (TD; IF; 40; G1). They also highlighted the relevance of the quality of communication between the different professionals and different teams:

“I think we should all sit to the table for a meeting. We now have a situation in our team in which all the decisions are made jointly, we have established: «When do we leave?», «When does the other team come in?». (…) Now we have even had to take a few steps back, but it has all gone beautifully because we are all in tune with each other…” (TD; IF; 42; G1).

According to several participants, the quality of the communication avoids the overlapping of functions with regards to the intervention—“(…) always speaking together to avoid overlapping someone else’s work so that we don’t duplicate tasks (…) We have to use our resources (…) that’s how it makes sense” (TD; IRC; 36; G1); “[during the intervention] we can even err by speaking a lot [to the children] because there are many of us and we should be coordinated to avoid confusion” (TD; IRC; 44; G1).

***Post-reunification team.*** All the professionals emphasized the idea that the success of family reunification requires an intervention team that can support the family after reunification has occurred:

“I think its success will depend on the support offered after the child comes home (…) a team to work with the family on a number of issues is necessary. (…) Over a long period of time there is a lot of support, and if they suddenly stop feeling supported (...) when we stop intervening, it all falls apart” (ED; IRC; 37; G4).

This team of reference for both the family and child could be an essential support basis to assure a balanced transition between foster care and family reunification, thus contributing to an adaptive family trajectory—“(…) a program [in the sense of a plan] with a formal close follow-up (..) not in the sense of inspection and control, but one of support” (TD; IRC; 43; G3).

#### 3.3.5. Sufficient Time between the Reunification Decision and the Child’s Re-Entry into the Family Home

With regards to the court’s family reunification decision, all the professionals revealed a critical position concerning the mandatory immediacy to reintegrate the child within their family, pointing to the need for a flexible length of time until the child’s re-entry in the family home—“We can’t have tight deadlines, we’re making serious decisions!” (TD; IF; 37; G2); “I think the court ought to be confronted with what we’re doing and sometimes it’s better to take an extra month to ensure it goes well rather than to simply deliver (…)” (TD; IF; 56; G1). This could contribute to an intervention “(…) with quality so that the child (…) may be gradually integrated into his/her own family” (TD; IF; 42; G2).

Although, according to the professionals, it is crucial to avoid the delay of decisions during the process, a tight and stiff temporal window does not leave room for the necessary intervention:

“When someone comes and says «look, the child’s leaving now», in other words, there’s been a sudden court order (…) We need time to work, to check if there’s an adequate response or not, but the court order comes so suddenly that we can’t” (ED; IRC; 30; G4).

Most of the professionals also underlined the need for a pre-reunification period, similar to the pre-adoption phase, corresponding with the first months of the child’s return to the family home:

“preparing them upon return… as in the monitoring for adoption: the pre-adoption phase [after the child’s integration in the adoptive family and before the legal adoption process has been finalized]. I think it’s important that the children don’t feel the abrupt cut and know that there’s a member of the team present to support them at this moment” (ED; IRC; 29; G4).

### 3.4. Dynamic Interaction among the Factors Influencing the Success of Reunification

**Figure 2:**
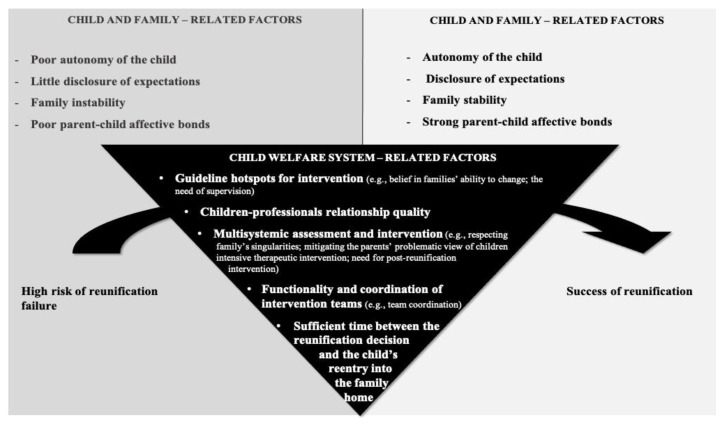
Dynamic Interactions Among Factors for Family Reunification Success.

A more in-depth analysis than the thematic analysis presented on the previous topics—grounded in interpreting the relationship among the main themes within the three systemic levels—led to the schematic construction of a systematization proposal to explain the dynamic process of the factors influencing the success of family reunification (Figure 2).

This interactive in-depth analysis revealed the participants’ dichotomic view on the child- and family-related factors regarding their influence on the reunification process. As shown in Figure 2, poor autonomy of the child, little disclosure of expectations, family instability, and poor parent–child affective bonds are associated with a high risk of reunification failure, while the inverse factors are related to a successful reunification process. The considerable importance attributed to child-welfare-system-related factors should be noted, which emerged as a powerful vehicle to convert child and family vulnerabilities into competencies. Hence, this interactive holistic analysis clearly points to the child welfare system as the scaffold for a path towards a successful reunification process.

## 4. Discussion

Through qualitative methodology, this study sought to explore and analyze the standpoint of child protection professionals with regards to the factors influencing successful reunification on the basis of their own experience.

The results shed light upon a set of influential factors within three different systemic levels: child, family, and child welfare system, which is in line with prior literature [29,30,31,33] and confirms the complexity of the reunification process [12].

The data analysis showed a clear predominance of influential factors associated with the child welfare system when compared with factors related to the child and family, which could suggest professionals’ strong conviction of the powerful role of the intervention system as a vehicle for recovery and change to further successful reunification trajectories.

Within the child welfare system, i.e., the intervention system, the results pointed to the relevance of five factors: Guideline Hotspots for Intervention; Children–Professionals Relationship Quality; Multisystemic Assessment and Intervention; Functionality and Coordination of Intervention Teams, and Sufficient Time between the Reunification Decision and the Child’s Re-Entry into the Family Home.

With regards to the Guideline Hotspots for Intervention, the child’s best interests emerged as a meta-guide, as was also consensually recommended by the scientific literature [20,39,51]. The participants also clearly emphasized the importance of believing in the families’ ability to change and in their competencies and strengths, as this contributes to a growing sense of self-confidence, thus reinforcing their self-organization capacity [64]. The need to define and implement a consistent Life Project for children, always with their best interests and needs at heart, and grounded on their idiosyncratic life contexts and family history, also emerged as a relevant guideline, and is considered an essential part of recovery [18,20,30,46]. Finally, in line with the scientific literature [18,20,55], the need for continuous supervision was highlighted as a key factor to assure the quality of assessment and intervention practices.

The results suggest that the quality of the Children–Professionals Relationship is a strong influential factor for a successful reunification process as it benefits the quality of communication throughout all its phases and fosters new learning in adjusted and healthy relational patterns between adults and children; moreover, several authors have also pointed to the therapeutic impact of the children–professionals’ positive relationship on insecure and disorganized attachment patterns commonly found in children in foster care [5,6,7].

The third theme to emerge—Multisystemic Assessment and Intervention—included several factors that characterize Multisystemic Therapy [65,66]. Within the scope of this theme, the professionals mentioned the need for: collaborative intervention; respecting families’ singularity; an early and comprehensive assessment and therapeutic intervention; and post-reunification intervention.

The data analysis revealed that the powerful influence of collaborative intervention on successful reunification [25,30,53,66] requires a positive relationship between professionals and families, respect for each family’s uniqueness [49,50], and, as already mentioned, a genuine conviction of the family’s competencies and strengths [64]. As stated by the professionals, collaborative intervention enhances parents’ view of intervention as a life changing opportunity and increases family motivation and involvement in the reunification process [18,29,40,42].

Early and comprehensive assessment emerged as a particularly relevant feature that could inform decisions about the child’s Life Project, and could be used to guide and nurture the comprehensive and intensive therapeutic intervention [65,66], which should also begin at the earliest stage possible [18,39]; moreover, it also serves to enrich the information reports forwarded to the court, thus contributing to the judges’ informed and balanced decisions, which reduces the risk of unsuccessful reunification trajectories. The emerging idea of the professional role as a scaffold for the transformation of child and family weaknesses into strengths is particularly noteworthy, thereby highlighting the welfare care system as a key resource for promoting the development and growth of families and children in family reunification processes [18,31,35].

According to the professionals, and in line with several authors [18,21,30,43,45], continuity of the intervention throughout the post-reunification phase, i.e., after the reintegration of the child into the family home, is a sine qua non condition that can assure the success of the reunification, thus preventing the child’s re-entry into the care system. It was even argued that the post-reunification intervention should be mandatory and bear no costs for families, as it is a guarantee of continued support so they are able to cope with and solve emerging problems during this period [17,18,51], which fosters the effective success of the family’s reunification [18,31,35].

With regards to the functionality and coordination of the intervention teams (foster care team, expert team on family intervention, and post-reunification team)—the fourth theme within the care system level that emerged through the data analysis—the results point to its relevance during the reunification process. As stated by the professionals, these different teams work with children and/or with families in the recovery and change processes and also have a crucial supportive role both in the child’s foster placement and after re-entry into their families. Therefore, and in line with the scientific literature, it was highlighted that clear boundaries and the coordinated work of different professionals and teams involved in each reunification process promotes the rigorous exchange of information, the quality of communication, and the continuity of a collaborative context, thus increasing the likelihood of the reunification’s success [18,25,30,42,53,54].

The results also revealed another influential factor within the care system level, which is clearly related to the legal system, as the professionals emphasized the need for a flexible length of time between the reunification decision and the child’s re-entry into the family home. In line with several authors [18,21,31,35], the professionals also exhibited a strong critical position towards the mandatory reintegration of children within their family immediately after the court decision, as this precludes the intervention to prepare the children and their families for the effective reality of reunification.

Within the child systemic level, only two main influential factors emerged—the Child’s Autonomy and Disclosure of Expectations—thus pointing to a more relational than psychopathological perspective, which contrasts with previous studies that have mentioned the predictive role of children’s emotional and behavioural problems in unsuccessful reunification [21,29,30,35,36]. A possible explanation for this divergence is the difference in the research methodology, since most of the afore-mentioned studies used a quantitative method and explored information on databases and/or data from children’s records. Child’s Autonomy and Disclosure of Expectations suggest the relevance of intervening with children to promote their well-being and maturity and help to prepare them for family reunification, as stated by Farmer [20].

With regards to the family systemic level, the results evidenced two meaningful influential factors: Family Stability and Parent–Child Affective Bonds. According to Kimberly et al. [31], family instability in terms of structure, social relationships, and life goals appears to be associated with parents’ ambivalence in their parental role. In this regard, and considering the relevance of the above-mentioned comprehensive assessment, professionals must be attentive to signs of family instability so that they can intervene as early as possible. As far as Parent–Child Affective Bonds are concerned, the professionals referred to their powerful role in the success of the reunification process and reinforced the importance of parents’ ability to develop such bonds. In this regard, as also suggested by Farmer [20], interactive and relational child–family moments, particularly in the “real” home context, should be increased, even if technical support is required.

The professionals highlighted the importance of comprehensive, integrated, and progressive intervention with families and children, thus corroborating the relevance of child and/or family preparation for reunification, which has been strongly recommended by several authors [19,20,25,40,44].

### Limitations and Strengths

Although the present study contributes to the deepening of knowledge on intervention in the family reunification process, it has some limitations that need to be acknowledged. The participants’ recruitment was based on the need for good informants with first-hand experiences in the reunification processes—child protection professionals, considering their diversity, i.e., residential care technical and educational workers, and professionals of specialized teams in family preservation and reunification—which contributes to a further understanding of professionals’ experiences and views of the family reunification intervention process. However, it should be noted that most of the participants were from two major urban regions: Porto, in the North Region, and Lisbon, in the Central Region of Portugal; most of them were female; and the sample was too broad as far as age and length of professional experience are concerned. Therefore, future studies considering the purpose of sampling ideas rather than sampling people [58,59,60] should consider wide-ranging geographic samples, thereby increasing the number of focus groups and accounting for the diversity of participants’ characteristics in order to expand the scope of relevant experiences and valuable insights on the reunification process, and to obtain a more robust study with regards to the theoretical saturation point [59]. Nevertheless, the qualitative analysis, through a continuous comparison process of data and interpretations and considering the approximation to the theoretical saturation point, allowed us to identify key common themes across the participants’ narratives on the reunification process.

It should also be mentioned that the qualitative nature of this study prevents total suppression of the researchers’ subjectivity. Notwithstanding, some efforts were made to minimize the interference of subjectivity following the basic requirements recommended by the grounded theory methodology [59]: the continuous use of reflectivity to guarantee a critical position of the main researcher towards the analysis, i.e., ongoing self-scrutiny regarding the influence of her interpretations and decisions, mainly through memo writing and regular meetings with the research team to discuss the analysis process; and the presentation and discussion of preliminary results with groups of psychology researchers, students, and professionals of the children’s care system.

Despite these limitations, several strengths can be highlighted: the qualitative analysis followed by main grounded theory requirements, namely, as mentioned above, continuous comparison and reflectivity [58,60]; this qualitative study is grounded on professionals’ voices on the family reunification process, including professionals with educational duties which, to our knowledge, are frequently unheard within the scope of literature on the theme. This provides useful information on reunification in Portugal, where research on the subject is scarce. Hence, this study provides evidence-based knowledge on the family reunification process that may enrich intervention practices, thus contributing to its success.

## 5. Conclusions

The present study revealed the meaningful and active role played by the professionals working in the child care system in the success of family reunification and which alerts public policies to the need to invest in professional resources and their technical training and supervision.

It should also be emphasized that the results revealed the relevance of early intervention, which should continue throughout post-reunification to assure the reinforcement and maintenance of balanced family relationships and the child’s psychological adjustment and well-being, thus promoting the success of the reunification process; moreover, to ensure a smooth and healthy transition between the foster placement and re-entry into the family, particular attention should be given to the preparation of the child for family reintegration. The relevance of the collaborative relationship among all those involved—professionals–families, professionals–child, and professionals–professionals—is another core indication for reunification practices.

Finally, this study points to the importance of parents’ close, positive, and collaborative contact with the child social care services as well as with their own placed children, which also suggests, particularly in the context of Portugal, the need for supportive public policies that not only drive families but also drive with and alongside them towards successful family reunification.

## Figures and Tables

**Table 1:** Key issues that foster the success of family reunification.

Systemic Level	Theme
Child	Child’s Autonomy
	Disclosure of Expectations
Family	Family Stability
	Parent–Child Affective Bonds
Child welfare system	Guideline Hotspots for Intervention Children–Professionals Relationship Quality
	Multisystemic Assessment and Intervention
	Functionality and Coordination of Intervention Teams
	Sufficient Time between the Reunification Decision and the Child’s Re-Entry into the Family Home

## Data Availability

Not applicable.

## References

[1] Landers A.L., Danes S.M. (2016). Forgotten Children: A Critical Review of the Reunification of American Indian Children in the Child Welfare System. Child Youth Serv. Rev..

[2] JBS International (2017). Children’s Bureau Child and Family Services Reviews: Aggregate Report.

[3] Debowska A., Willmott D., Boduszek D., Jones A.D. (2017). What Do We Know about Child Abuse and Neglect Patterns of Co-Occurrence? A Systematic Review of Profiling Studies and Recommendations for Future Research. Child Abus. Negl..

[4] Liming K.W., Grube W.A. (2018). Wellbeing Outcomes for Children Exposed to Multiple Adverse Experiences in Early Childhood: A Systematic Review. Child Adolesc. Soc. Work. J..

[5] Bovenschen I., Lang K., Zimmermann J., Förthner J., Nowacki K., Roland I., Spangler G. (2016). Foster Children’s Attachment Behavior and Representation: Influence of Children’s Pre-Placement Experiences and Foster Caregiver’s Sensitivity. Child Abus. Negl..

[6] Cyr C., Euser E.M., Bakermans-Kranenburg M.J., van Ijzendoorn M.H. (2010). Attachment Security and Disorganization in Maltreating and High-Risk Families: A Series of Meta-Analyses. Dev. Psychopathol..

[7] Vorria P., Papaligoura Z., Dunn J., van Ijzendoorn M.H., Steele H., Kontopoulou A., Sarafidou Y. (2003). Early Experiences and Attachment Relationships of Greek Infants Raised in Residential Group Care. J. Child Psychol. Psychiatry.

[8] Teixeira D.N., Silva S.R., Henriques M.R. (2018). Crianças Em Acolhimento Residencial: Conteúdo Temático Das Suas Narrativas de Vida. [Children in Foster Care: Thematic Content of Their Life Narratives]. Anál. Psicol..

[9] Howe M.L., Cicchetti D., Toth S.L. (2006). Children’s Basic Memory Processes, Stress, and Maltreatment. Dev. Psychopathol..

[10] Dansey D., John M., Shbero D. (2018). How Children in Foster Care Engage with Loyalty Conflict: Presenting a Model of Processes Informing Loyalty. Adopt Foster.

[11] Broadhurst K., Mason C. (2017). Birth Parents and the Collateral Consequences of Court-Ordered Child Removal: Towards a Comprehensive Framework. Int. J. Law Policy Fam..

[12] Balsells M.A., Pastor C., Mateos A., Vaquero E., Urrea A. (2015). Exploring the Needs of Parents for Achieving Reunification: The Views of Foster Children, Birth Family and Social Workers in Spain. Child Youth Serv. Rev..

[13] del Valle J.F., del Valle J., Bravo A. (2009). Cómo Potenciar La Reunificación Familiar Desde Los Centros y Hogares de Protección. Intervención Socieducativa en Acogimiento Residencial.

[14] Fernandez E., Lee J.-S. (2013). Accomplishing Family Reunification for Children in Care: An Australian Study. Child Youth Serv. Rev..

[15] López M., del Valle J.F., Montserrat C., Bravo A. (2013). Factors Associated with Family Reunification for Children in Foster Care. Child Fam. Soc. Work.

[16] Balsells M.A., Pastor C., Molina M., Fuentez-Pelaez N., Vaqueiro E., Mundet A. (2013). Child Welfare and Successful Reunification: Understanding of the Famiy Difficulties during the Socio-Educative Process. Revista de Cercetare si Interventie Sociala.

[17] Huscroft-D’Angelo J., Trout A., Epstein M., Duppong-Hurley K., Thompson R. (2013). Gender Differences in Perceptions of Aftercare Supports and Services. Child Youth Serv. Rev..

[18] Carlson L., Hutton S., Priest H., Melia Y. (2020). Reunification of Looked-after Children with Their Birth Parents in the United Kingdom: A Literature Review and Thematic Synthesis. Child Fam. Soc. Work.

[19] Farmer E. (2014). Improving Reunification Practice: Pathways Home, Progress and Outcomes for Children Returning from Care to Their Parents. Br. J. Soc. Work.

[20] Farmer E. (2018). Reunification from Out-of-Home Care: A Research Overview of Good Practice in Returning Children Home from Care.

[21] Font S.A., Sattler K.M.P., Gershoff E. (2018). When Home Is Still Unsafe: From Family Reunification to Foster Care Reentry. J. Marriage Fam..

[22] Huscroft-D’Angelo J., Trout A.L., Henningsen C., Synhorst L., Lambert M., Patwardhan I., Tyler P. (2019). Legal Professional Perspectives on Barriers and Supports for School-Aged Students and Families during Reunification from Foster Care. Child Youth Serv. Rev..

[23] Montserrat C. (2014). The Child Protection System from the Perspective of Young People: Messages from 3 Studies. Soc. Sci..

[24] Brown S.M., Orsi R., Chen P.C.B. (2019). Child, Family, and Case Characteristics Associated with Reentry into Out-of-Home Care Among Children and Youth Involved with Child Protection Services. Child Maltreat..

[25] Chambers J.M., Lint S., Thompson M.G., Carlson M.W., Graef M.I. (2019). Outcomes of the Iowa Parent Partner Program Evaluation: Stability of Reunification and Re-Entry into Foster Care. Child Youth Serv. Rev..

[26] Cheng T.C. (2010). Factors Associated with Reunification: A Longitudinal Analysis of Long-Term Foster Care. Child Youth Serv. Rev..

[27] Courtney M.E. (1994). Factors Associated with the Reunification of Foster Children with Their Families. Soc. Serv. Rev..

[28] Farmer E., Wijedasa D. (2013). The Reunification of Looked After Children with Their Parents: What Contributes to Return Stability?. Br. J. Soc. Work.

[29] Carnochan S., Rizik-Baer D., Austin M.J. (2013). Preventing Re-Entry to Foster Care. J. Evid. Based Soc. Work.

[30] Jedwab M., Shaw T.V. (2017). Predictors of Reentry into the Foster Care System: Comparison of Children with and without Previous Removal Experience. Child Youth Serv. Rev..

[31] Kimberlin S.E., Anthony E.K., Austin M.J. (2009). Re-Entering Foster Care: Trends, Evidence, and Implications. Child Youth Serv. Rev..

[32] Li D., Chu C.M., Ng W.C., Leong W. (2014). Predictors of Re-Entry into the Child Protection System in Singapore: A Cumulative Ecological–Transactional Risk Model. Child Abus. Negl..

[33] Jones A.S., LaLiberte T. (2017). Risk and Protective Factors of Foster Care Reentry: An Examination of the Literature. J. Public Child Welf..

[34] Mc Grath-Lone L., Dearden L., Nasim B., Harron K., Gilbert R. (2016). Changes in First Entry to Out-of-Home Care from 1992 to 2012 among Children in England. Child Abus. Negl..

[35] Davidson R.D., Tomlinson C.S., Beck C.J., Bowen A.M. (2019). The Revolving Door of Families in the Child Welfare System: Risk and Protective Factors Associated with Families Returning. Child Youth Serv. Rev..

[36] Lutman E., Farmer E. (2013). What Contributes to Outcomes for Neglected Children Who Are Reunified with Their Parents? Findings from a Five-Year Follow-up Study. Br. J. Soc. Work.

[37] Parolini A., Shlonsky A., Magruder J., Eastman A.L., Wulczyn F., Webster D. (2018). Age and Other Risk Factors Related to Reentry to Care from Kin Guardian Homes. Child Abus. Negl..

[38] Esposito T., Caldwell J., Chabot M., Delaye A., Trocmé N., Hélie S., Fallon B. (2021). Reunification Trajectories in Quebec: Acknowledging Chronic Family Challenges to Support Stability. Child Abus. Negl..

[39] Wulczyn F. (2004). Family Reunification. Future Child.

[40] Wells M., Correia M. (2012). Reentry into Out-of-Home Care: Implications of Child Welfare Workers’ Assessments of Risk and Safety. Soc. Work Res..

[41] LaBrenz C.A., Fong R., Cubbin C. (2020). The Road to Reunification: Family- and State System-Factors Associated with Successful Reunification for Children Ages Zero-to-Five. Child Abus. Negl..

[42] Arizmendi J., Almeida A. (2017). Reunificação Familiar e Acolhimento Residencial Em Portugal-Norte: Visões Dos Intervenientes. [Family Reunification and Residential Care in Portugal-North: Views of the Stakeholders]. Revista de Estudios e Investigación en Psicología y Educación.

[43] Shipe S.L., Shaw T.V., Betsinger S., Farrell J.L. (2017). Expanding the Conceptualization of Re-Entry: The Inter-Play between Child Welfare and Juvenile Services. Child Youth Serv. Rev..

[44] Biehal N. (2006). Reuniting Children with Their Families: Reconsidering the Evidence on Timing, Contact and Outcomes. Br. J. Soc. Work.

[45] Child Welfare Information Gateway (2017). Supporting Successful Reunifications.

[46] Murphy A.L., Harper W., Griffiths A., Joffrion C. (2017). Family Reunification: A Systematic Review of Interventions Designed to Address Co-Occurring Issues of Child Maltreatment and Substance Use. J. Public Child Welf..

[47] U.S. Department of Health (2000). Framework for the Assessment of Children in Need and Their Families.

[48] Bronfenbrenner U. (1986). Ecology of the Family as a Context for Human Development. Research Perspectives. Dev. Psychol..

[49] Balsells M.A.B., Mateops A.I., Urrea-Monclús A.U., Vaquero E.T. (2018). Positive Parenting Support during Family Reunification. Early Child Dev. Care.

[50] Osterling K.L., Han M. (2011). Reunification Outcomes among Mexican Immigrant Families in the Child Welfare System. Child Youth Serv. Rev..

[51] James S.L., Roby J.L., Powell L.J., Teuscher B.A., Hamstead K.L., Shafer K. (2017). Does Family Reunification from Residential Care Facilities Serve Children’s Best Interest? A Propensity-Score Matching Approach in Ghana. Child Youth Serv. Rev..

[52] Kemp S.P., Marcenko M.O., Lyons S.J., Kruzich J.M. (2014). Strength-Based Practice and Parental Engagement in Child Welfare Services: An Empirical Examination. Child Youth Serv. Rev..

[53] Lee S.Y., Benson S.M., Klein S.M., Franke T.M. (2015). Accessing Quality Early Care and Education for Children in Child Welfare: Stakeholders’ Perspectives on Barriers and Opportunities for Interagency Collaboration. Child Youth Serv. Rev..

[54] Phillips J.D. (2022). A Mixed Methods Study of How Interprofessional Communication Relates to Timely Reunification in the U.S. Child Welfare System. J. Public Child Welf..

[55] Arbeiter E., Toros K. (2017). Participatory Discourse: Engagement in the Context of Child Protection Assessment Practices from the Perspectives of Child Protection Workers, Parents and Children. Child Youth Serv. Rev..

[56] Instituto da Segurança Social, I.P. (2021). CASA 2020: Relatório de Caracterização Anual Da Situação de Acolhimento Das Crianças e Jovens.

[57] Marshall C., Rossman G. (1989). Designing Qualitative Research.

[58] Birks M., Millls J. (2011). Grounded Theory—A Practical Guide.

[59] Charmaz K. (2006). Constructing Grounded Theory: A Practical Guide through Qualitative Analysis.

[60] Daly K.J. (2007). Qualitative Methods for Family Studies and Human Development.

[61] Percy W., Kostere K., Kostere S. (2015). Generic Qualitative Research in Psychology. Qual. Rep..

[62] Braun V., Clarke V. (2006). Using Thematic Analysis in Psychology. Qual. Res. Psychol..

[63] Maguire S.A., Williams B., Naughton A.M., Cowley L.E., Tempest V., Mann M.K., Teague M., Kemp A.M. (2015). A Systematic Review of the Emotional, Behavioural and Cognitive Features Exhibited by School-Aged Children Experiencing Neglect or Emotional Abuse. Child Care Health Dev..

[64] Ausloos G. (2003). A Competência Das Famílias [The Ability of Families].

[65] Henggeler S.W., Schoenwald S.K., Borduin C.M., Rowland M.D., Cunningham P.B. (2009). Multisystemic Therapy for Antisocial Behavior in Children and Adolescents.

[66] Swenson C.C., Schaeffer C.M. (2018). A Multisystemic Approach to the Prevention and Treatment of Child Abuse and Neglect. Int. J. Child Maltreat..

